# A Review on Carrier Mobilities of Epitaxial Graphene on Silicon Carbide

**DOI:** 10.3390/ma16247668

**Published:** 2023-12-15

**Authors:** Wataru Norimatsu

**Affiliations:** Faculty of Science and Engineering, Waseda University, Tokyo 169-8555, Japan; norimatsu@waseda.jp

**Keywords:** graphene, SiC, mobility, carrier density

## Abstract

Graphene growth by thermal decomposition of silicon carbide (SiC) is a technique that produces wafer-scale, single-orientation graphene on an insulating substrate. It is often referred to as epigraphene, and has been thought to be suitable for electronics applications. In particular, high-frequency devices for communication technology or large quantum Hall plateau for metrology applications using epigraphene are expected, which require high carrier mobility. However, the carrier mobility of as-grown epigraphene exhibit the relatively low values of about 1000 cm^2^/Vs. Fortunately, we can hope to improve this situation by controlling the electronic state of epigraphene by modifying the surface and interface structures. In this paper, the mobility of epigraphene and the factors that govern it will be described, followed by a discussion of attempts that have been made to improve mobility in this field. These understandings are of great importance for next-generation high-speed electronics using graphene.

## 1. Introduction

In 2004, Novoselov, Geim et al. reported the electronic properties of graphene as a new two-dimensional electron system [1]. They revealed that the carrier mobility of graphene reached 10,000 cm^2^/Vs at the carrier density of 1 × 10^13^ cm^−2^. In the next year, they demonstrated the quantum Hall effect using graphene [2]. In parallel, in 2004, de Heer et al. reported that graphene epitaxially grown on a silicon carbide (SiC) substrate also behaves as a two-dimensional electron system [3]. In 2006, it was reported that graphene on SiC exhibited a mobility of 25,000 cm^2^/Vs at 3.4 × 10^12^ cm^−2^ [4].

Graphene is often seen as the ultimate two-dimensional material with a thickness of one atom and has been actively researched as one of the main players in two-dimensional electron systems. The electronic band structure of graphene has been investigated theoretically since the middle of the 20th century [5,6]. It has linear band dispersion around the K point in reciprocal space, which results in a high Fermi velocity. The linear band dispersion of graphene was observed experimentally through angle-resolved photoemission spectroscopy [7].

Following the graphene fever ignited by Geim et al., there were various reports on the electronic properties of graphene that impact its applications. In particular, semiconductor applications that utilize high mobility, which were investigated in the early stages, attracted much attention. Since graphene essentially has surfaces on both sides, it was thought that external factors such as adsorbents and contact with the substrate and electrodes would have a large influence on its mobility. Indeed, in 2008, a mobility of 200,000 cm^2^/Vs at an electron density of 2 × 10^11^ cm^−2^ was reported by using suspended graphene to remove the substrate-derived effects [8]. On the other hand, in practical terms, it is necessary to prepare graphene on a substrate. By sandwiching graphene with hexagonal boron nitride (h-BN), a two-dimensional insulator, and making edge contact with the electrode, a carrier mobility of 140,000 cm^2^/Vs was reported at 2 × 10^11^ cm^−2^ [9]. The development of high-carrier-mobility graphene has progressed rapidly in the past twenty years, and there are game changers each year. For example, many attempts have been made to obtain high mobility, such as single-crystal-like graphene grown on polycrystalline copper foil exhibiting 10,000 cm^2^/Vs [10], graphene directly grown on SiO_2_/Si substrate exhibiting 9000 cm^2^/Vs [11], monolayer graphene sandwiched by two layers of CrOCl insulator exhibiting 540,000 cm^2^/Vs [12], and potassium-doped nanographene exhibiting 3000 cm^2^/Vs [13]. When considering further applications, a technology for growing graphene directly on an insulating substrate is essential. In this review article, I will mainly focus on epitaxial graphene grown on SiC, which is available as an insulating or wide-gap semiconductor substrate. Epitaxial graphene on SiC was given the name “epigraphene” by de Heer et al. [14], and I will use this term in this review.

**Figure 1:**
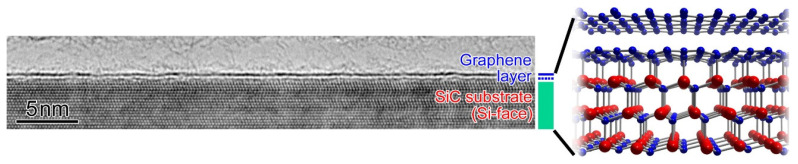
High-resolution TEM image of epigraphene on SiC, together with its structural model [15].

Epigraphene is formed on a SiC substrate by thermal decomposition. This has the particular advantage that electronic devices can be fabricated using commercially available semi-insulating SiC wafers without the need for a physical transfer of graphene. Figure 1 shows a transmission electron microscope (TEM) image and a schematic diagram of epigraphene [15]. It has an interface layer called a buffer layer between the graphene and SiC, which is shown by a dotted line in the figure [16]. Although the in-plane atomic arrangement of the buffer layer is almost the same as that of graphene, some carbon atoms are strongly bonded to the silicon atoms just below, and so it does not exhibit the properties of freestanding graphene.

It was clearly stated in 1896 that graphite was formed by the thermal decomposition of SiC [17]. Later, in the middle of the 20th century, X-ray diffraction and electron diffraction experiments revealed the layered growth of graphite on SiC [18,19]. In 1998, it was reported that a single layer of graphite was formed on the surface of hexagonal 6H-SiC (0001) [20]. In 2008, it was reported that wafer-scale, large-area epigraphene growth is possible when SiC single crystals are heated in an atmospheric pressure of Ar [21,22]. Many review papers and books have been published regarding the growth, structure, and properties of epigraphene [23,24,25,26,27,28,29,30]. Now, a technique for the growth of wafer-scale single-oriented epigraphene on an insulating substrate has been established. For this reason, epigraphene has an extremely superior advantage in electronics applications of graphene.

Since 2004, graphene’s high mobility has been expected to be used in semiconductor applications. However, it has become apparent that since there is no bandgap, the on/off ratio is small, making it difficult to apply to digital logic devices. Based on this background, epigraphene is expected to have two major applications. One is in analog high-frequency transistors for next-generation information and communication devices. Such high-frequency devices using epigraphene have been reported to have a cutoff frequency of 300 GHz and an oscillation frequency of 70 GHz [31,32,33,34]. Current silicon devices have a physical limit of a few tens of gigahertz. However, due to heat generation issues, they can actually be operated only at 3–4 GHz. Therefore, high-frequency transistors based on epigraphene are promising. The other is in metrology applications. In quantum Hall measurements using epigraphene, a much wider quantum Hall plateau was observed compared to materials such as GaAs [35,36,37,38]. This allows epigraphene to be used as a quantum resistance standard. To this end, high-quality graphene growth technology is being developed. In both applications, it is important that the carriers in graphene can transport at high speed. In this review article, I will explain the results from the previous research on the mobility of epigraphene.

## 2. Basics of the Mobility of Epigraphene

**Figure 2:**
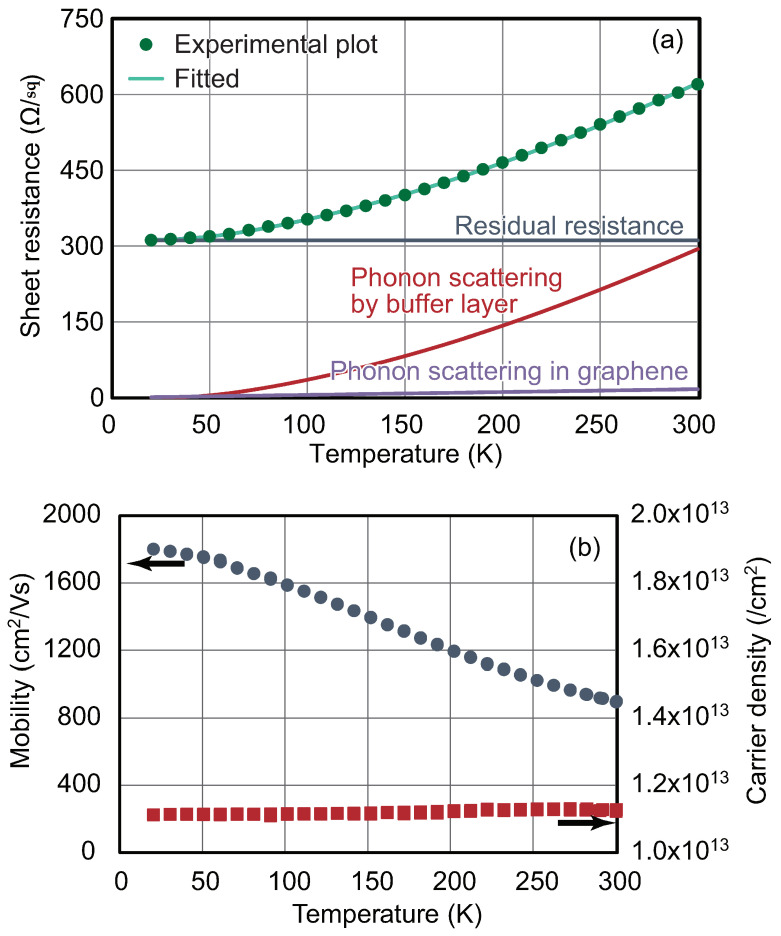
Electrical properties of monolayer epigraphene on SiC. Temperature dependence of the (**a**) sheet resistance and (**b**) carrier mobility (blue circles) and carrier density (red squares) [30]. In (**a**), solid lines are the results of fitting based on the equation shown in the text.

Figure 2 shows the results of the Hall effect measurements on a typical epigraphene sample [15,30]. In Figure 2a, the temperature dependence of sheet resistance is shown. The resistance increases with increasing temperature, indicating typical metallic or semimetallic behavior. The sheet resistance at room temperature (RT) is approximately 600 Ω/sq. In Figure 2b, the blue circle and the red square plots show the temperature dependence of the mobility and the carrier density. The carrier type is electrons, and the electron density is almost independent of temperature and is in the order of 10^13^ cm^−2^. On the other hand, the mobility decreases with increasing temperature and is approximately 900 cm^2^/Vs at RT. These behaviors are understood by the fact that the electronic state of graphene is characterized as a semimetal. The temperature dependence of mobility and sheet resistance is dominated by carrier scattering by phonons. It is known that the sheet resistance of epigraphene can be expressed as follows based on Matthiessen’s law [39,40,41,42]:

(1) $$R=R_{0}+R_{LAP}+R_{IP}$$

Here, *R*_0_ is the residual resistance due to defects and impurities, which is not temperature-dependent. The quantities *R*_LAP_ and *R*_IP_ are the resistance due to the longitudinal acoustic phonons in graphene and to the remote interfacial phonons, respectively. *R*_LAP_ can be expressed by

(2) $$R_{LAP}=\frac{\pi D_{A}^{2}k_{B}}{e^{2}\hbar \rho_{s}v_{s}^{2}v_{F}^{2}}T$$

where *D*_A_ is the deformation potential in graphene, *k*_B_ is the Boltzmann constant, *e* is the electron charge, ℏ is the Dirac constant, $\rho_{s}$ is the two-dimensional mass density of graphene, vs. is the sound velocity, and *v*_F_ is the Fermi velocity. And *R*_IP_ is expressed by the following equation:

(3) $$R_{IP}=\sum_{i=1}^{2}\left\{\frac{C_{i}}{\exp\left(\frac{E_{i}}{k_{B}T}\right)-1}\right\}$$

where *C_i_* is the coefficient of the electron–phonon coupling, and *E_i_* is the corresponding phonon energy. Here, the values *E*_1_ = 70 and *E*_2_ = 16 meV are used, which correspond to the phonon energies of the buffer layer. The plots in Figure 2a can be fitted by these equations. The fitting parameters are *D_A_* = 14 eV, *C*_1_ = 994 Ω, *C*_2_ = 191 Ω, and *R*_0_ = 311 Ω [30]. The above results indicate that most of the resistance at low temperatures is due to residual resistance, and that the resistance increase with increasing temperature is derived from interfacial phonons. That is, the thermal vibration of carbon atoms in the buffer layer scatters electrons in the graphene on top of it. In other words, controlling both the carrier density and the interface structure, including the presence of the buffer layer, is quite important for the electronic state and physical properties of graphene. Interface engineering is one of the critical keywords in epigraphene research.

From here, I explain the factors that affect the mobility in epigraphene. The mobility at RT of 900 cm^2^/Vs at a carrier density of 1 × 10^13^ cm^−2^ is comparable to that of Si, but it is somewhat lower than the mobility of graphene mentioned in the introduction. Theoretically, the mobility of graphene on SiC at RT is predicted to reach 100,000 cm^2^/Vs at 1 × 10^12^ cm^−2^ when considering the phonons in graphene, the polar phonons on the SiC substrate surface, and charged impurities [43,44]. Furthermore, it is known that the mobility in graphene is proportional to 1/√*n* with respect to the carrier density *n* [41,43]. Therefore, at 1 × 10^13^ cm^−2^, there should be a potential mobility of about 30,000 cm^2^/Vs at RT. The reasons for the lower mobility observed compared to the theoretical predictions are the following:

Graphene quality;
Substrate effects;
Interface effects.

These three factors are discussed in the following sections.

### 2.1. Graphene Quality

The quality of a graphene sample can be divided into chemical and physical aspects, which correspond to the presence of impurities and defects, respectively. The results of the Hall effect measurements described above showed that the carrier density was as high as 10^13^ cm^−2^. However, this is mainly due to the polarization of the SiC substrate and does not necessarily mean that there are many impure elements. Since hexagonal SiC has spontaneous polarization, it is understood that epigraphene is electron-doped to about 1 × 10^13^ cm^−2^ due to surface charging and the presence of a buffer layer [45]. It is possible to modulate the carrier density by modifying the interface structure using a technique like intercalation, which will be discussed later.

Concerning the physical aspects, probing the number of defects is possible using Raman spectroscopy measurements. It is known that in a typical Raman spectrum of epigraphene, the D band is quite small, indicating that there are few defects [15,30]. It is also known that local non-uniformity in the number of graphene layers, such as a mixture of monolayer and bilayer regions, can become a scattering center [46]. Even when a monolayer and bilayer coexist, it is possible to analyze the mobility within each region by analyzing the magnetoresistance. In such a case, at 10 mK, mobilities of 20,000 cm^2^/Vs at 1.9 × 10^12^ cm^−2^ in a monolayer region and 1400 cm^2^/Vs at 5.1 × 10^12^ cm^−2^ in a bilayer region were reported [47]. These chemical and physical factors collectively affect the mobility of epigraphene.

By exfoliating graphene from a SiC substrate and transferring it onto another substrate, it is possible to assess the quality of the epigraphene. Such attempts have actually been made, and the highest mobility of graphene transferred from SiC onto SiO_2_ was 7500 cm^2^/Vs at 5 × 10^11^ cm^−2^ at RT [48,49]. But in this case, graphene might well be damaged in the transfer process.

### 2.2. Substrate Effects

As mentioned above, the polarization of the hexagonal SiC substrate directly affects the carrier density [45]. On the other hand, the influence of doping in the SiC substrate is a technical aspect. In order to perform electrical conductivity measurements on epigraphene, SiC needs to be an insulating substrate. Semi-insulating SiC substrates are actually commercially available. Semi-insulating SiC mainly includes high-purity semi-insulating (HPSI) SiC and V-doped semi-insulating SiC. As SiC manufacturers, CREE and TankeBlue sell HPSI SiC and II-VI (now Coherent) sells V-doped semi-insulating SiC. Even in the same hexagonal SiC, 4H- and 6H-SiC exist as polytypes, and the Si-terminated (0001) and C-terminated (0001) surfaces exist as polar surfaces. Note that the carrier type is electrons in all samples.

**Figure 3:**
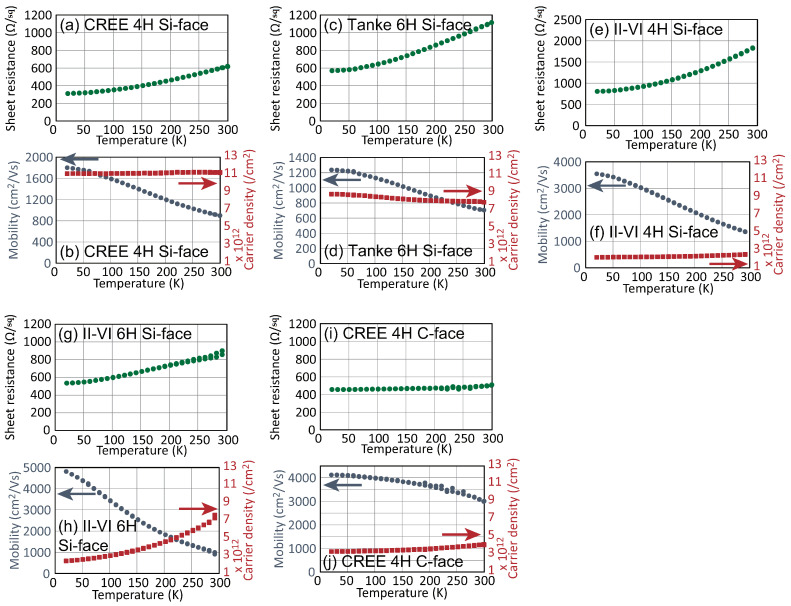
Sheet resistance, mobility, and carrier density of epigraphene on different SiC substrates [30]. The substrates are (**a**,**b**) CREE 4H-SiC (0001), (**c**,**d**) TankeBlue 6H-SiC (0001), (**e**,**f**) II-VI 4H-SiC (0001), (**g**,**h**) II-VI 6H-SiC (0001), and (**i**,**j**) CREE 4H-SiC (0001). In (**b**,**d**,**f**,**h**,**j**), circles and squares are mobility and carrier density, respectively.

Figure 3 shows the results of Hall effect measurements made using epigraphene on these substrates [30]. In the figure, experimental results using Figure 3a,b 4H-SiC (0001) HPSI from CREE, which are the same as in Figure 2; Figure 3c,d 6H-SiC (0001) HPSI from TankeBlue; Figure 3e,f V-doped 4H-SiC (0001) from II-VI; Figure 3g,h V-dope 6H-SiC (0001) from II-VI; and Figure 3i,j 4H-SiC (0001) HPSI from CREE substrates are shown. Similar to Figure 2, the common features of epigraphene on the Si-terminated (0001) surface in Figure 3a–h are that the sheet resistance increases, and the mobility decreases with increasing temperature. Here, in epigraphene on HPSI SiC in Figure 3b,d, the electron density is about 8–11 × 10^12^ cm^−2^, while in epigraphene on V-doped SiC in Figure 3f,h, the electron density is about 2 × 10^12^ cm^−2^ at low temperatures. In particular, in Figure 3h, the electron density increased with increasing temperature. These results suggest that HPSI and V-doped SiC have different impurity levels, which affect the electron density and its temperature dependence. Epigraphene on the V-doped semi-insulating substrate has the highest mobility due to its low electron density. In any case, in epigraphene on the SiC (0001) surface, the mobility decreases with increasing temperature, so the resistance also increases. The sheet resistance at RT ranges from 600 to 1500 Ω/sq.

On the other hand, epigraphene on C-terminated SiC (0001) exhibited different features. Unlike the Si-terminated surface, the temperature dependence of mobility is small. As a result, the sheet resistance also has a small temperature dependence, and its value at RT is about 500 Ω/sq.

**Figure 4:**
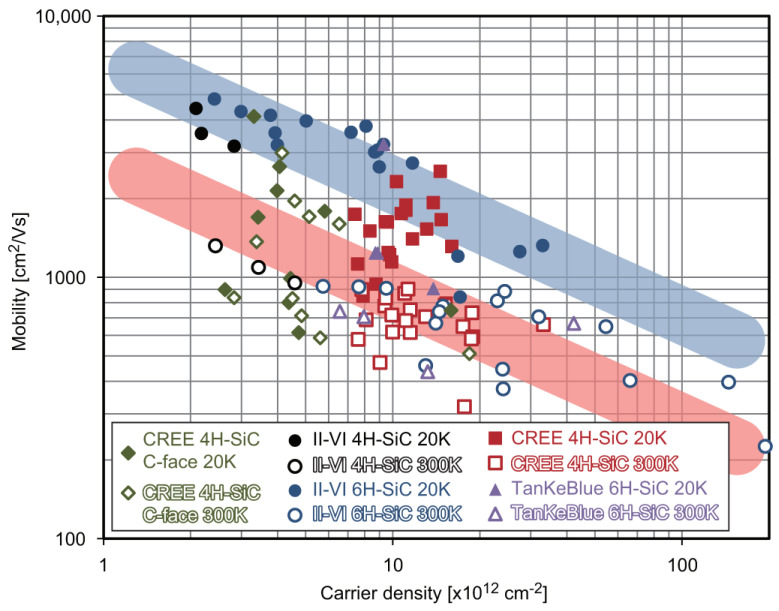
Carrier mobility versus carrier density of epigraphene on various SiC substrates [30]. Closed and open symbols of the same color refer, respectively to the mobility at 20 and 300 K of the samples displayed using the same outline color. Blue and red lines are guides to the eye based on the μ∝1/√n relation.

There are variations in mobility and carrier density between samples. Figure 4 shows the mobility of graphene samples grown using these types of SiC substrates plotted against carrier density [30]. In the figure, the mobility values at 20 and 300 K are shown as closed and open circles, respectively. The green diamonds are from graphene on the C-terminated (0001) surface, and the others are from graphene on the Si-terminated (0001) surface. The mobility distribution differs for each substrate type. The overall trend is that the mobility increases as the carrier density decreases. The 20 and 300 K plots appear to roughly follow the blue and red lines. The slopes of these straight lines are assumed to be μ∝1/√n. Graphene on V-doped SiC has the highest mobility. At RT, graphene on the C-face has the highest mobility, but its values greatly vary. This is due to the fact that while it is easy to control the number of graphene layers on the Si-face, it is difficult to control it on the C-face. On the C-face, there is a distribution of the number of layers, and the rotational relation between graphene layers is relatively random [50,51]. The rotational relation of multilayer graphene on the C-face has been under discussion since 2011, and recently it has been suggested that it may be controlled by the off-angle of the SiC substrate [52,53,54].

As mentioned in the previous sections, substrate polar phonons also have a large effect on mobility, particularly at RT. However, the mobility is about an order of magnitude lower than theoretical predictions that take these surface phonons into account, so other contributions are therefore larger. Irregularities such as steps existing on the SiC substrate surface have a large impact on the mobility [55,56]. The resistance is higher in the direction beyond the step, and the higher the step height, the greater the resistance [57,58]. It is known that graphene growth involves high-temperature heating, which causes step bunching and increases the height of the steps [59]. Techniques to control step bunching on SiC surfaces prior to [60] and during [61] graphene growth have been reported. In addition, Si vacancies in the vicinity of SiC surface were reported, since the thermal decomposition of SiC accompanies Si desorption [62]. These are also considered to be carrier scattering sources. Recently, it has been reported that the resistance of adjacent terraces differs depending on the type of Si-C bilayer on the surface of hexagonal SiC, which is induced by the surface energy difference [63]. These substrate-induced factors have a combined effect on the mobility of epigraphene.

### 2.3. Interface Effect

The interface between graphene and the SiC substrate includes a buffer layer. Electrons in graphene are scattered by phonons in the buffer layer. Therefore, the mobility decreases as the temperature increases. This suggests that the electrical conduction mechanism can be modified by controlling the interface structure. The buffer layer can be transformed into graphene by intercalating hydrogen or other species at the interface [64]. Hydrogen can cut the bonds between the carbon atoms in the buffer layer and the silicon atoms in the SiC, and saturate silicon dangling bonds. As will be described later, by performing hydrogen intercalation, it is possible to suppress the mobility decrease due to temperature rise, and the mobility at RT is approximately 3000 cm^2^/Vs [65]. It has been reported that the buffer layer can be transformed into graphene by intercalation of various elements or physical treatments, and its electronic state can be significantly modulated [66,67].

## 3. Improving the Mobility of Epigraphene

**Figure 5:**
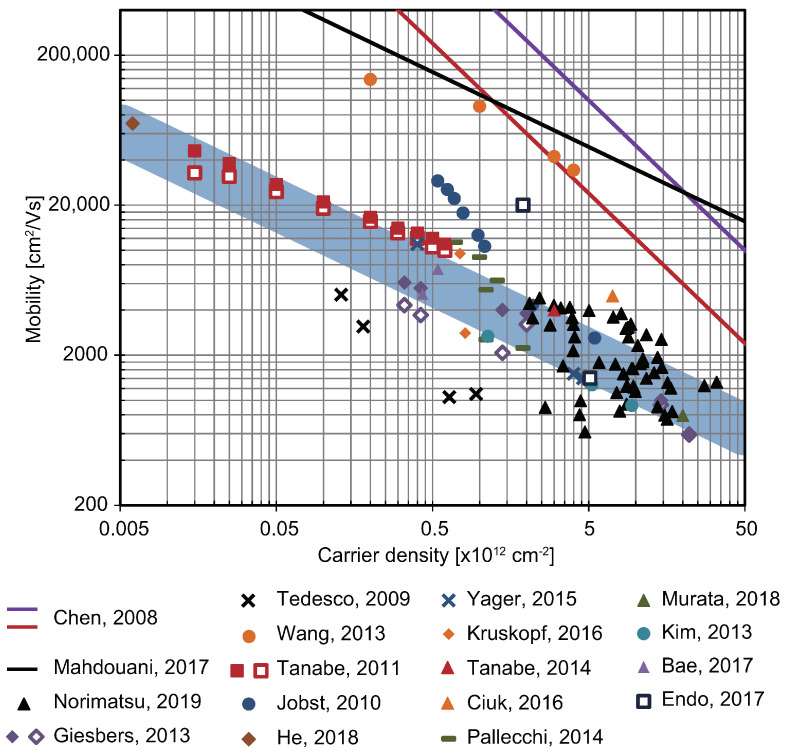
Mobility versus carrier density of epigraphene reported in the literature [30]. Plots are from the references shown in the bottom of the figure [9,30,41,42,48,49,69,70,71,72,73,74,75,76]. Black, purple, and red lines are theoretical mobility limits [39,44]. The blue line is a guide to the eye based on the μ∝1/√n relationship.

The carrier mobility of epigraphene is affected by various factors. In other words, by modifying these factors, mobility can be improved. Figure 5 shows the relation between the mobility and the carrier density of epigraphene that has been reported. In the figure, the plots indicated by the black triangles are the same as the values of as-grown epigraphene at 20 K shown in Figure 4 [30]. Mobility values based on the typical Hall effect measurements are also plotted as purple diamonds and black crosses [42,68]. The red and purple lines are the theoretical limits at 300 and 77 K when considering only phonons in graphene [39]. The black line is the theoretical limit at RT when polar phonons in SiC are considered [44]. The orange circles indicate the mobility of graphene sandwiched by h-BN, rather than graphene on SiC [9].

The most direct method to improve the mobility of graphene is to control the carrier density. The fact that the mobility is proportional to 1/√*n* with respect to the carrier density *n* indicates that the mobility can be improved by lowering the carrier density, for which there are physical and chemical methods available. In terms of physical methods, carrier density can be electrically tuned in the so-called field-effect transistors [40,41]. This technique enabled the mobility to increase up to 46,000 cm^2^/Vs at 2 K and a carrier density of 1.5 × 10^10^ cm^−2^ to be obtained, which are shown by red squares [41]. The highest mobility in epigraphene was obtained via a chemical approach. The adsorption of acceptor molecules with high electron affinity onto graphene resulted in hole doping [69,70]. A mobility of 70,000 cm^2^/Vs at 2 K, 0.6 × 10^10^ cm^−2^ was reported when an appropriate concentration of F4TCNQ molecules was blended into a polymer and coated on epigraphene, which is shown by a brown diamond [70]. Although not shown in Figure 5, the adsorption of water molecules on epigraphene has also been shown to reduce electron density and to slightly increase mobility [77,78]. It has also been reported that when graphene was treated with ozone under water, the carrier density became 4 × 10^10^ cm^−2^ and the mobility at 2 K became 11,000 cm^2^/Vs, as shown by blue crosses [71]. The fact that a mobility of tens of thousands was experimentally obtained through carrier density reduction shows the high potential of epigraphene. This is very important for high-frequency electronics and metrology applications. On the other hand, it is still lower than that of graphene sandwiched by h-BN, indicating that the full potential of graphene has not yet been exploited just by the carrier density reduction.

Mobility can also be improved by modifying the structure of the surface or interface. As for the surface morphology, it is expected that mobility and its isotropy will increase by suppressing the step bunching that accompanies graphene growth. It was shown that applying a thin layer of polymer to the SiC surface and then performing thermal decomposition at relatively low temperatures forms a buffer layer, suppressing step bunching. This technique is called polymer-assisted sublimation growth (PASG), which, for graphene on SiC with a step height of 0.25 nm, resulted in a mobility of 9500 cm^2^/Vs at 2.2 K and 7.5 × 10^11^ cm^−2^, which is shown by orange diamonds [72].

As for interface modification, the most typical method is hydrogen intercalation at the interface. After growing a uniform buffer layer, hydrogen can be intercalated by heating in a hydrogen atmosphere, and then the buffer layer turns into graphene, the data for which are shown by red and orange triangles in Figure 5 [64,65,73,74,79]. As a result, a maximum mobility of 11,300 cm^2^/Vs at 7 × 10^11^ cm^−2^ has been reported, as shown by green bars above [75]. In hydrogen intercalation, hydrogen permeates through the buffer layer to reach the interface [80]. If Si dangling bonds that are not terminated with hydrogen remain, they become a scattering center and reduce mobility [76]. Therefore, it is important that all Si atoms are terminated with hydrogen atoms. Intercalation of other elements was widely reported, but the mobility was not high. Among them, oxygen is a relatively small atom, and epigraphene treated with oxygen had only a low mobility of around 790 cm^2^/Vs, which is shown by a green triangle [81]. This is because defects are introduced into graphene during the intercalation process.

As mentioned in Section 2.1, epigraphene can be peeled off from the SiC substrate in order to completely eliminate interface and substrate effects [48,49]. The highest mobility found in this case was 7500 cm^2^/Vs at RT and 5 × 10^11^ cm^−2^, which is shown by purple triangles [49]. Mobility values, which are extracted from the analysis of magnetoresistance, are also plotted as blue squares, although they deviated slightly from the overall trend [47]. This result indicates that the extreme uniformity of monolayer graphene is quite important. As a completely different approach, research has been reported in which electrons are confined in one dimension by forming graphene into a nanoribbon shape. Generally, nanoribbonization has been performed to introduce a bandgap into graphene [82]. On the other hand, ballistic conduction was achieved in a graphene nanoribbon by utilizing nanofacets on the SiC surface [83]. Although the mobility cannot be defined in ballistic conduction, it was reported in the literature that when the value was converted to mobility it was 6,000,000 cm^2^/Vs at 4 K. All of these techniques may be important in high-speed electronics applications.

Table 1 summarizes the results of the high mobility of epigraphene, which is particularly important in Figure 5. Overall, mobility is most strongly dependent on carrier density. As-grown epigraphene has a relatively high carrier density. In addition, mobility changes due to various factors shown in Section 2.1, Section 2.2 and Section 2.3. For example, plots located far below the thick lines in Figure 4 and Figure 5 indicate that the quality of the graphene may be significantly poor. In other words, plots located on a thick line can be considered to represent graphene of the same quality. This indicates that this graph could serve as a benchmark for graphene quality. For instance, if as-grown graphene, which is shown by black triangles, is processed in some way and its mobility moved onto this thick line, it can be understood that the quality of the graphene remains the same, and only the carrier density has changed. Conversely, if it changes to below the thick line, it means that the quality of the graphene has deteriorated.

The same can be said about interfaces. For example, if hydrogen intercalation is applied to graphene, and the mobility plot initially located below the thick line moves directly above the line, it shows that the mobility has improved due to the interface effect, independent of the carrier density. On the other hand, when the substrate is changed by transferring graphene, the story is different. The thick line is based on the mobility of epigraphene on SiC, so in principle it reflects the physical properties of SiC, such as surface polar phonons. Therefore, even if the mobility is on a thick line, as in the plots of [48,49] after being transferred onto a SiO_2_/Si substrate, it cannot be said that there was no change in the quality of graphene. That is, it cannot indicate that there is no damage caused by the transfer process. In any case, when comparing mobility values, it is necessary to comprehensively understand the carrier density, graphene quality, substrate effect, interface effect, and so on. Temperature is another important factor, and the mobility at 20 K is approximately twice that at RT, which is estimated from Figure 4. In addition, the device fabrication process also affects device performance in actual applications. In this manuscript, the results of only a typical FET device were described. Device fabrication includes a lot of aspects, and the doping process also affects the performance. However, improving mobility remains important for applications.

## 4. Future Directions

In a nutshell, graphene is characterized by high carrier mobility, but no bandgap. This makes it ideal for analog high-frequency electronics as described in the introduction. The mobility enhancement techniques described in the previous section and their combination would further expand the possibilities for these applications. For example, extremely high mobility was reported for structures in which graphene is sandwiched between h-BN [9]. If this can be combined with large-area graphene growth technology on SiC substrates, i.e., h-BN/graphene/h-BN/SiC heterostructures, higher mobility may be achieved. Although such attempts have been made, very high mobility has not been realized so far [84,85,86,87]. A larger-scale and higher-quality heterostructure together with the carrier density reduction is required.

In high-frequency electronics using epigraphene, cutoff frequencies of 300 GHz and oscillation frequency of 70 GHz have been reported [31,32,33,34]. On the other hand, if just aiming for high frequency, up to about 700 GHz has already been reported using InP [88,89]. In the future, 1 THz technology is desired. By combining the techniques described in this paper, it is expected that devices operating at a higher frequency, such as 1 THz, would possibly be achieved. Improvements in the device structure would also help. These would lead to the development of next-generation communication technologies for the ever-increasing mobile communication traffic in the future.

## 5. Conclusions

In this article, I reviewed the carrier mobility in epigraphene on SiC. The mobility of graphene generally depends on carrier density and temperature. In addition to these factors, the mobility of epigraphene is significantly modulated by the substrate and interface structure. In other words, the mobility can be improved by controlling these factors. The typical relation of $\mu \propto 1/\sqrt{n}$ shown in this paper can be used as a benchmark for measuring the quality of epigraphene. Improving mobility is an extremely important factor, especially for high-frequency transistor applications.

## Figures and Tables

**Table 1:** Comparison table of the carrier density and mobility ranges of epigraphene, which are extracted from Figure 5 as representative values. For comparison, the value of a CVD-grown graphene is also listed.

Type	Carrier Density [cm^−2^]	Mobility [cm^2^/Vs]	Ref. #
F4TCNQ	6 × 10^9^	70,000	[70]
FET	1.5 × 10^10^–6 × 10^11^	46,000–11,000	[41]
Magnetoresistance	1.9 × 10^12^	20,000	[47]
Hydrogen intercalation	7 × 10^11^–1.9 × 10^12^	11,300–2200	[75]
PASG	8 × 10^11^	9500–2800	[72]
Ozone	4 × 10^11^–4.6 × 10^12^	11,000–1400	[71]
Transferred	4.3 × 10^11^–5.4 × 10^11^	7500–5100	[49]
As-grown	2.1 × 10^12^–1.4 × 10^14^	4800–400	[30]
CVD graphene	2 × 10^11^–4 × 10^12^	137,600–34,200	[9]

## Data Availability

No new data were created or analyzed in this study. Data sharing is not applicable to this article.

## References

[1] Novoselov K.S., Geim A.K., Morozov S.V., Yiang D., Zhang Y., Dubonos S.V. (2004). Electric Field Effect in Atomically Thin Carbon Films. Science.

[2] Novoselov K.S., Geim A.K., Morozov S.V., Jiang D., Katsnelson M.I., Grigorieva I.V., Dubonos S.V., Firsov A.A. (2005). Two-dimensional gas of massless Dirac fermions in graphene. Nature.

[3] Berger C., Song Z.M., Li T.B., Li X.B., Ogbazghi A.Y., Feng R., Dai Z.T., Marchenkov A.N., Conrad E.H., First P.N. (2004). Ultrathin epitaxial graphite: 2D electron gas properties and a route toward graphene-based nanoelectronics. J. Phys. Chem. B.

[4] Berger C., Song Z., Li X., Wu X., Brown N., Naud C., Mayou D., Li T., Hass J., Marchenkov A.N. (2006). Electronic Confinement and Coherence in Patterned Epitaxial Graphene. Science.

[5] Wallace P.R. (1947). The band theory of graphite. Phys. Rev..

[6] Castro Neto A.H., Guinea F., Peres N.M.R., Novoselov K.S., Geim A.K. (2009). The electronic properties of graphene. Rev. Mod. Phys..

[7] Bostwick A., Ohta T., Seyller T., Horn K., Rotenberg E. (2007). Quasiparticle dynamics in graphene. Nature.

[8] Bolotin K.I., Sikes K.J., Jiang Z., Klima M., Fudenberg G., Hone J., Kim P., Stormer H. (2008). Ultrahigh electron mobility in suspended graphene. Solid State Commun..

[9] Wang L., Meric I., Huang P.Y., Gao Q., Gao Y., Tran H., Taniguchi T., Watanabe K., Campos L.M., Muller D.A. (2013). One-Dimensional Electrical Contact to a Two-Dimensional Material. Science.

[10] Vlassiouk I.V., Stehle Y., Pudasaini P.R., Unocic R.R., Rack P.D., Baddorf A.P., Ivanov I.N., Lavrik N.V., List F., Gupta N. (2018). Evolutionary selection growth of two-dimensional materials on polycrystalline substrates. Nat. Mater..

[11] Tyagi A., Miseikis V., Martini L., Forti S., Mishra N., Gebeyehu Z.M., Giambra M.A., Zribi J., Fregnaux M., Aureau D. (2022). Ultra-clean high-mobility graphene on technologically relevant substrates. Nanoscale.

[12] Zhang Y., Wang S., Hu G., Huang H., Zheng B., Zhou Y., Feng Y., Ma X., He J., Lu Y. (2022). Giant carrier mobility if graphene with enhanced Shubnikov-de-Haas quantum oscillations: Implications for low-power-consumption device applications. ACS Appl. Nano Mater..

[13] Yamada T., Masuzawa T., Okigawa Y. (2023). Patassium-doped nano graphene as an intermediate layer for graphene electronics. Appl. Phys. Lett..

[14] Berger C., Conrad E.H., de Heer W.A. (2018). Epigraphene: Epitaxial graphene on silicon carbide. Physics of Solid Surfaces, Subvolume B.

[15] Kusunoki M., Norimatsu W., Bao J., Morita K., Starke U. (2015). Growth and Features of Epitaxial Graphene on SiC. J. Phys. Soc. Jpn..

[16] Norimatsu K., Kusunoki M. (2009). Transitional structures of the interface between graphene and 6H–SiC (0001). Chem. Phys. Lett..

[17] Acheson E.G. (1896). U.S. Patent.

[18] Badami D.V. (1962). Graphitization of α-Silicon Carbide. Nature.

[19] Van Bommel A.J., Crombeen J.E., van Tooren A. (1975). LEED and Auger electron observations of the SiC(0001) surface. Surf. Sci..

[20] Forbeaux I., Themlin J.M., Debever J.M. (1998). Heteroepitaxial graphite on 6H-SiC(0001): Interface formation through conduction-band electronic structure. Phys. Rev. B.

[21] Emtsev K.V., Bostwick A., Horn K., Jobst J., Kellogg G.L., Ley L., McChesney J.L., Ohta T., Reshanov S.A., Rotenberg E. (2009). Towards wafer-size graphene layers by atmospheric pressure graphitization of silicon carbide. Nat. Mater..

[22] Virojanadara C., Syväjarvi M., Yakimova R., Johansson L.I. (2008). Homogeneous large-area graphene layergrowth on 6H-SiC(0001). Phys. Rev. B.

[23] De Heer W.A., Berger C., Wu X., Sprinkle M., Hu Y., Ruan M., Stroscio J.A., First P.N., Haddon R., Piot B. (2010). Epitaxial graphene electronic structure and transport. J. Phys. D Appl. Phys..

[24] Riedl C., Coletti C., Starke U. (2010). Structural and electronic properties of epitaxial graphene on SiC(0 0 0 1): A review of growth, characterization, transfer doping and hydrogen intercalation. J. Phys. D Appl. Phys..

[25] Hibino H., Tanabe S., Mizuno S., Kageshima H. (2012). Growth and electronic transport properties of epitaxial graphene on SiC. J. Phys. D Appl. Phys..

[26] Norimatsu K., Kusunoki M. (2014). Structural features of epitaxial graphene on SiC {0001} surfaces. J. Phys. D Appl. Phys..

[27] Mishra N., Boeckl J., Motta N., Iacopi F. (2016). Graphene growth on silicon carbide: A review. Phys. Status Solidi A.

[28] Yazdi G.R., Iakimov T., Yakimova R. (2016). Epitaxial Graphene on SiC: A Review of Growth and Characterization. Crystals.

[29] Pradeepkumar A., Gaskill D.K., Iacopi F. (2020). Electronic and Transport Properties of Epitaxial Graphene on SiC and 3C-SiC/Si: A Review. Appl. Sci..

[30] Norimatsu W., Terasawa T., Matsuda K., Bao J., Kusunoki M. (2019). Features and Prospects for Epitaxial Graphene on SiC. Handbook of Graphene.

[31] Lin Y.M., Dimitrakopoulos C., Jenkins K.A., Farmer D.B., Chiu H.-Y., Grill A., Avouris P. (2010). 100-GHz Transistors from Wafer-Scale Epitaxial Graphene. Science.

[32] Avouris P., Xia F. (2012). Graphene applications in electronics and photonics. MRS Bull..

[33] Yu C., He Z.Z., Li J., Song X.B., Liu Q.B., Cai S.J., Feng Z.H. (2016). Quasi-free-standing bilayer epitaxial graphene field-effect transistors on 4H-SiC (0001) substrates. Appl. Phys. Lett..

[34] Guo Z., Dong R., Chakraborty P.S., Lourenco N., Palmer J., Hu Y., Ruan M., Hankinson J., Kunc Y., Cressler J.D. (2013). Record Maximum Oscillation Frequency in C—Face Epitaxial Graphene Transistors. Nano Lett..

[35] Tzalenchuk A., Lara-Avila S., Kalaboukhov A., Paolillo S., Syväjärvi M., Yakimova R., Kazakova O., Janssen T.J.B.M., Fal’ko V., Kubatkin S. (2010). Towards a quantum resistance standard based on epitaxial graphene. Nat. Nanotechnol..

[36] Janssen T.J.B.M., Tzalenchuk A., Lara-Avila S., Kubatkin S., Fal’ko V.I. (2013). Quantum resistance metrology using graphene. Rep. Prog. Phys..

[37] Janssen T.J.B.M., Rozhko S., Antonov I., Tzalenchuk A., Williams J.M., Melhem Z., He H., Lara-Avila S., Kubatkin S., Yakimova R. (2015). Operation of graphene quantum Hall resistance standard in a cryogen-free table-top system. 2D Mater..

[38] Kruskopf M., Elmquist R.E. (2018). Epitaxial graphene for quantum resistance metrology. Metrologia.

[39] Chen J.-H., Jang C., Xiao S., Ishigami M., Fuhrer M.S. (2008). Intrinsic and extrinsic performance limits of graphene devices on SiO_2_. Nat. Nanotechnol..

[40] Zhu W., Perebeinos V., Freitag M., Avouris P. (2009). Carrier scattering, mobilities, and electrostatic potential in monolayer, bilayer, and trilayer graphene. Phys. Rev. B.

[41] Tanabe S., Sekine Y., Kageshima H., Nagase M., Hibino H. (2011). Carrier transport mechanism in graphene on SiC (0001). Phys. Rev. B.

[42] Giesbers A., Procházka P., Flipse C. (2013). Surface phonon scattering in epitaxial graphene on 6H-SiC. Phys. Rev. B.

[43] Perebeinos V., Avouris P. (2010). Inelastic scattering and current saturation in graphene. Phys. Rev. B.

[44] Mahdouani M. (2017). Investigation of the electron-surface phonon interaction effects in graphene on a substrate made of polar materials. Phys. E.

[45] Ristein J., Mammadov S., Seyller T. (2012). Origin of doping in quasi-free-standing graphene on silicon carbide. Phys. Rev. Lett..

[46] Chua C., Connolly M., Lartsev A., Yager T., Lara-Avila S., Kubatkin S., Kopylov S., Fal’ko V., Yakimova R., Pearce R. (2014). Quantum Hall Effect and Quantum Point Contact in Bilayer-Patched Epitaxial Graphene. Nano Lett..

[47] Endo A., Bao J., Norimatsu W., Kusunoki M., Katsumoto S., Iye Y. (2017). Two-carrier model on the magnetotransport of epitaxial graphene containing coexisting single-layer and bilayer area. Philos. Mag..

[48] Kim J., Park H., Hannon J.B., Bedell S.W., Fogel K., Sadana D.K., Dimitrakopoulos C. (2013). Layer-resolved graphene transfer via engineered strain layers. Science.

[49] Bae S.H., Zhou X., Kim S., Lee Y.S., Cruz S.S., Kim Y., Hannon J.B., Yang Y., Sadana D.K., Ross F.M. (2017). Unveiling the carrier transport mechanism in epitaxial graphene for forming wafer-scale, single-domain graphene. Proc. Natl. Acad. Sci. USA.

[50] Creeth G.L., Strudwick A.J., Sadowski J.T., Marrows C.H. (2011). Surface morphology and transport studies of epitaxial graphene on SiC (000-1). Phys. Rev. B.

[51] Norimatsu W., Takada J., Kusunoki M. (2011). Formation mechanism of graphene layers on SiC (000-1) in a high-pressure argon atmosphere. Phys. Rev. B.

[52] Mathieu C., Barrett N., Rault J., Mi Y.Y., Zhang B., de Heer W.A., Berger C., Conrad E.H., Renault O. (2011). Microscopic correlation between chemical and electronic states in epitaxial graphene on SiC (000-1). Phys. Rev. B.

[53] Johansson L., Watcharinyanon S., Zakharov A.A., Iakimov T., Yakimova R., Virojanadara C. (2011). Stacking of adjacent graphene layers grown on C-face SiC. Phys. Rev. B.

[54] Sakakibara R., Bao J., Hayashi N., Ito T., Hibino H., Norimatsu W. (2023). Control of rotation angles of multilayer graphene on SiC (000-1) by substrate off-direction and angle. J. Phys. Condens. Matter.

[55] Low T., Perebeinos V., Tersoff J., Avouris P. (2012). Deformation and scattering in graphene over substrate steps. Phys. Rev. Lett..

[56] Dimitrakopoulos C., Grill A., McArdle T.J., Liu Z., Wisnieff R., Antoniadis D.A. (2011). Effect of SiC wafer miscut angle on the morphology and Hall mobility of epitaxially grown graphene. Appl. Phys. Lett..

[57] Ji S.H., Hannon J.B., Tromp R.M., Perebeinos V., Tersoff J., Ross F.M. (2012). Atomic-scale transport in epitaxial graphene. Nat. Mater..

[58] Sinterhauf A., Traeger G.A., Pakdehi D., Pierz K., Schumacher H.W., Wenderoth M. (2021). Unravelling the origin of local variations in the step resistance of epitaxial graphene on SiC: A quantitative scanning tunneling potentiometry study. Carbon.

[59] Oliveira M.H., Schumann T., Ramsteiner M., Lopes J.M.J., Riechert H. (2011). Influence of the silicon carbide surface morphology on the epitaxial graphene formation. Appl. Phys. Lett..

[60] Sakakibara R., Bao J., Yuhara K., Matsuda K., Terasawa T., Kusunoki M., Norimatsu W. (2023). Step unbunching phenomenon on 4H-SiC (0001) surface during hydrogen etching. Appl. Phys. Lett..

[61] Bao J., Yasui O., Norimatsu W., Matsuda K., Kusunoki M. (2016). Sequential control of step-bunching during graphene growth on SiC (0001). Appl. Phys. Lett..

[62] Gruschqitz M., Schletter H., Schulze S., Alexandrou I., Tegenkamp C. (2019). Epitaxial graphene on 6H-SiC(0001): Defects in SiC investigated by STEM. Phys. Rev. Mater..

[63] Sinterhauf A., Traeger G.A., Pakdehi D.M., Schadlich P., Speck F., Seyller T., Tegenkamp C., Pierz K., Schumacher H.W., Wenderoth M. (2020). Substrate induced nanoscale resistance variation in epitaxial graphene. Nat. Commun..

[64] Riedl C., Coletti C., Iwasaki T., Zakharov A.A., Starke U. (2009). Quasi-Free-Standing Epitaxial Graphene on SiC Obtained by Hydrogen Intercalation. Phys. Rev. Lett..

[65] Speck F., Jobst J., Fromm F., Ostler M., Waldmann D., Hundhausen M., Weber H.B., Seyller T. (2011). The quasi-free-standing nature of graphene on H-saturated SiC(0001). Appl. Phys. Lett..

[66] Rosenzweig P., Karakachian H., Marchenko D., Kuster K., Starke U. (2020). Overdoping graphene beyond the van Hove singularity. Phys. Rev. Lett..

[67] Bao J., Norimatsu W., Iwata H., Matsuda K., Ito T., Kusunoki M. (2016). Synthesis of freestanding graphene on SiC by a rapid-cooling technique. Phys. Rev. Lett..

[68] Tedesco J.L., VanMil B.L., Myers-Ward R.L., McCrate J.M., Kitt S.A., Campbell P.M., Jernigan G.G., Culbertson J.C., Eddy C.R., Gaskill D.K. (2009). Hall effect mobility of epitaxial graphene grown on silicon carbide. Appl. Phys. Lett..

[69] Jobst J., Waldmann D., Speck F., Hirner R., Maude D.K., Seyller T., Weber H.B. (2010). Quantum oscillations and quantum Hall effect in epitaxial graphene. Phys. Rev. B.

[70] He H., Kim K.H., Danilov A., Montemurro D., Yu L., Park Y.W., Lombardi F., Bauch T., Moth-Poulsen K., Iakimov T. (2018). Uniform doping of graphene close to the Dirac point by polymer-assisted assembly of molecular dopants. Nat. Commun..

[71] Yager T., Webb M.J., Grennberg H., Yakimova R., Lara-Avila S., Kubatkin S. (2015). High mobility epitaxial graphene devices vis aqueous-ozone processing. Appl. Phys. Lett..

[72] Kruskopf M., Pakdehi D.M., Pierz K., Wundrack S., Stosch R., Dziomba T., Götz M., Baringhaus J., Aprojanz J., Tegenkamp C. (2016). Comeback of epitaxial graphene for electronics: Large-area growth of bilayer-free graphene on SiC. 2D Mater..

[73] Tanabe S., Takamura M., Harada Y., Kageshima H., Hibino H. (2014). Effects of hydrogen interactions on transport properties of quasi-free-standing monolayer graphene. Jpn. J. Appl. Phys..

[74] Ciuk T., Petruk O., Kowalik A., Jozwik I., Rychter A., Szmidt J., Strupinski W. (2016). Low-noise epitaxial graphene on SiC Hall effect element for commercial applications. Appl. Phys. Lett..

[75] Pallecchi E., Lafont F., Cavaliere V., Schopfer F., Mailly D., Poirier W., Ouerghi A. (2014). High electron mobility in epitaxial graphene on 4H-SiC(0001) via post-growth annealing under hydrogen. Sci. Rep..

[76] Murata Y., Cavallucci T., Tozzini V., Pavlicek N., Gross L., Meyer G., Takamura M., Hibino H., Beltram F., Heun S. (2018). Atomic and electronic structure of Si dangling bonds in quasi-free-standing monolayer graphene. Nano Res..

[77] Melios C., Winters M., Strupinski W., Panchal V., Giusca C.E., Imalka-Jayawardena K.D.G., Rorsman N., Ravi S., Silva P., Kazakova O. (2017). Tuning epitaxial graphene sensitivity to water by hydrogen intercalation. Nanoscale.

[78] Kitaoka M., Nagahama T., Nakamura K., Aritsuki T., Takashima K., Ohno Y., Nagase M. (2017). Carrier doping effect of humidity for single-crystal graphene on SiC. Jpn. J. Appl. Phys..

[79] Robinson J.A., Hollander M., LaBella M., Trumbull K.A., Cavalero R., Snyder D.W. (2011). Epitaxial graphene transistors: Enhancing performance via hydrogen intercalation. Nano Lett..

[80] Sakakibara R., Norimatsu W. (2022). Microscopic mechanism of hydrogen intercalation: On the conversion of the buffer layer on SiC to graphene. Phys. Rev. B.

[81] Ostler M., Fromm F., Koch R.J., Wehrfritz P., Speck F., Vita H., Bottcher S., Horn K., Seyller T. (2014). Buffer layer free graphene on SiC(0001) via interface oxidation in water vapor. Carbon.

[82] Han M.Y., Ozyilmaz B., Zhang Y., Kim P. (2007). Energy band-gap engineering of graphene nanoribbons. Phys. Rev. Lett..

[83] Baringhaus J., Ruan M., Edler F., Tejeda A., Sicot M., Taleb-Ibrahimi A., Li A.P., Jiang Z., Conrad E.H., Berger C. (2014). Exceptional ballistic transport in epitaxial graphene nanoribbons. Nature.

[84] Shin H.C., Jang Y., Kim T.H., Lee J.H., Oh D.H., Ahn S.J., Lee J.H., Moon Y., Park J.H., Yoo J.H. (2015). Epitaxial growth of a single-crystal hybridized boron nitride and graphene layer on a wide-band gap semiconductor. J. Am. Chem. Soc..

[85] Sediri H., Pierucci D., Hajilaoui M., Henck H., Patriarche G., Dappe Y.J., Yuan S., Toury B., Belkhou R., Silli M.G. (2015). Atomically sharp interface in an h-BN-epitaxial graphene van der Waals heterostructure. Sci. Rep..

[86] Rigosi A.F., Liu C.I., Glavin N.R., Yang Y., Mill H.M., Hu J., Walker A.R.H., Richter C.A., Elmquist R.E., Newell D.B. (2017). Electrical stabilization of surface resistivity in epitaxial graphene systems by amorphous boron nitride encapsulation. ACS Omega.

[87] Gigliotti J., Li X., Sundaram S., Deniz D., Prudkovskiy V., Turmaud J.P., Hu Y., Hu Y., Fossard F., Merot J.S. (2020). Highly ordered boron nitride-epigraphene epitaxial films on silicon carbide by lateral epitaxial deposition. ACS Nano.

[88] Schwierz F. (2010). Graphene transistors. Nat. Nanotechnol..

[89] Schwierz F. (2013). Graphene transistors: Status, prospects, and problems. Proc. IEEE.

